# “He Who Sings, Prays Twice”? Singing in Roman Catholic Mass Leads to Spiritual and Social Experiences That Are Predicted by Religious and Musical Attitudes

**DOI:** 10.3389/fpsyg.2020.570189

**Published:** 2020-09-17

**Authors:** Melanie Wald-Fuhrmann, Sven Boenneke, Thijs Vroegh, Klaus Peter Dannecker

**Affiliations:** ^1^Department of Music, Max Planck Institute for Empirical Aesthetics, Frankfurt/Main, Germany; ^2^Theological Faculty, Trier, Germany; ^3^German Liturgical Institute, Trier, Germany

**Keywords:** communal singing, group singing, social bonding, music and spirituality, Catholic worship, attitudes and behavior

## Abstract

Singing is an essential element in every religion. In the liturgy of the Roman Catholic Church, theologians expect congregational singing to have several clear-cut effects which can be translated into psychological hypotheses. This study is the first to approach these quantitatively. *N* = 1603 Catholics from German-speaking countries answered an exhaustive questionnaire that asked whether and to what degree these putative effects were actually experienced by churchgoers. We found that people do, to a large degree, associate feelings of community and spiritual experiences with congregational singing. We also identified relevant intraindividual factors that contribute to the frequency of these experiences, most importantly, religious and musical attitudes. These results are discussed in the light of psychological literature on the effects of group singing on social bonding and wellbeing, but also in the context of theological, ethnomusicological, and sociological research on singing, songs, and spiritual and social experiences.

## Introduction

Song and singing are paramount in the rituals of every known religion (Beck, 2006). It is performed by individuals, specific groups, and entire congregations and is expected to have a range of effects and functions that directly contribute to the efficaciousness of the ritual. This claim is especially valid for Christianity, with singing in both public and private worship having played a prominent role since the early Church (Gelineau, 1964; Page, 2010). The Apostle Paul’s injunction to sing psalms, hymns, and spiritual songs to God (Ephesians 5:19, Colossians 3:16) stands in this regard side by side with both Saint Augustine’s accounts of being deeply moved by liturgical hymns and songs (*Confessions* IX:6, 14 and X:33, 49; Fuhrmann, 2004, pp. 104–111) and the famous expression “Bis orat qui cantat” (“He who sings, prays twice”), which had been ascribed to him for a long time. After centuries of a primarily clerical liturgy in which the liturgical chants and hymns were performed by specialized ensembles (the schola or cappella), the assembly’s active role, particularly with regard to singing and the authorization of vernacular hymns, was reinstated, first by the Protestant denominations in the sixteenth century, then by the Roman Catholic Church after the Second Vatican Council of 1964–1968.

This reform was accompanied by theological arguments stressing the role of singing as a major expression of the active participation of the assembled congregants, which in turn was viewed as a pivotal component of the reformed Catholic mass (*Sacrosanctum Concilium* [SC] 14–20, 30; *Musicam sacram* [MS] 15s.; Chan, 1998; Haunerland, 2016). In these arguments, active participation “both internally and externally” (SC 19) is claimed to be not only beneficial (MS 10), but also “the primary and indispensable source from which the faithful are to derive the true Christian spirit” (SC 14) and a prerequisite for the actual “sanctification of man” that is believed to happen in and through Holy Mass (SC 10). Singing, in particular, is claimed to have a “ministerial function” that leads to specific psychological and spiritual effects (SC 112–121).

Given the high expectations the Catholic Church expresses concerning congregational singing in the renewed liturgy, one can only wonder that there has been almost no empirical research attempting to substantiate these claims and exploring whether and under what circumstances the desired effects are actually generated. Apparently, the Church simply takes these effects for granted. With this, it is in good company. For centuries, people have time and again reiterated their belief in the spiritual effects of sacred music and corroborated it with (proto-)psychological theories, historical examples, and fictional and autobiographical narratives (Bohlman et al., 2006; Boynton, 2009).

The kind of experiences liturgical singing in a Catholic Mass can actually and reliably afford is a question that must be of concern for the Church. However, it is also a relevant topic for academic disciplines such as hymnology or psychology of music or religion. Therefore, this paper takes up the idea of accompanying the liturgical reforms with empirical studies (SC 44; Searle, 1983; Collins, 1987; Foley, 1995) and presents a first quantitative empirical exploration of this topic by reporting the results of an online survey that asked German-speaking Catholics about their singing experiences during Holy Mass. Hypotheses concerning the types of experiences were derived from the Church’s theory of liturgical singing; thus, it was the main aim of this study to establish whether singing in Mass could elicit the effects expected by the Church. We aimed to see if—in terms of Moore and Myerhoff (1977, pp. 10–15)—the “doctrinal efficacy” of Catholic worship was also accompanied by “operational efficacy” such as could be experienced individually. Although due to the study design, we were not able to check for systematic relationships between the concrete forms and repertoires of singing and the reported singing experiences, we were nevertheless able to look for individual factors by also collecting personal data about sociodemographics as well as religious and musical practices and attitudes.

## Materials and Methods

### Theological Theories About Liturgical Singing

For the renewed form of Roman-Catholic liturgy, three main authoritative sources spell out the Church’s norms, theories, and hypotheses about congregational singing: *Sacrosanctum concilium* (SC, 1963), the central document by the Second Vatican Council on the reform of the liturgy; *Musicam sacram* (MS, 1967), the Council’s instructions on music in the reformed liturgy; and finally the *Institutio generalis missalis Romani* (IGMR, 1975, 2002), the introduction to the Missal, the book that is used in actual celebrations of Mass. Although these documents provide neither an explicit theory of liturgical singing nor a systematization of expected effects, it is possible to deduce something like these from discrete references to singing in them. In addition to the pragmatic, semiotic, and decorative functions of the chants and hymns, three types of psychological effects are expected: spiritual effects, social effects, and dispositional effects (Wald-Fuhrmann, 2020).

Given the religious character of Mass, spiritual effects are certainly the most important ones. They are circumscribed as “the sanctification of the faithful” (MS 4), the raising of minds to heavenly things or to God (MS 5, 15), and the embellishment of prayer (SC 112; MS 5). These effects can be defined as anagogical.

A second important topic closely related to the nature of liturgical worship is the social effects of communal singing, most importantly the fostering of unity among the hearts and minds of those gathered (SC 112; MS 5; IGMR 46, 86). In essence, communal singing should not only express the unity of the congregation, which is a central ecclesiological theorem, but also continually (re-)produce it.

A further topic of great importance is the enhancing effect song or music is believed to have on the words it accompanies. This close connection of music with the main liturgical parts of the Mass Proper and Ordinary is the primary justification for the “pre-eminence” that is given to music over any other art in Catholic worship (SC 112). The musical clothing of religious texts is supposed to direct the attention of the congregants to the words they sing and in this way to facilitate their effects on congregants’ minds and souls. People are expected not only to pay closer attention to a text when singing it, but to be enabled thus to make its meaning and emotional tone their own in order to become the “I” or “We” expressed in the chant or to perform various speech acts, such as acclaiming, praising, imploring, or professing (IGMR 52, 53, 62). This effect might be called the dispositional effect, and it consists mainly of persuasion and emotional contagion. It was already codified in an earlier document about church music, namely *Tra le sollecitudini* (TLS) by Pope Pius X (1903).

Taken together, these anagogical, unifying, and dispositional effects clearly attest to the critical functions attributed to songs and singing in Catholic worship today. For the sake of the present study, they can be translated into empirically testable hypotheses, namely: (1) Liturgical singing facilitates feeling connected to God (spiritual effect); (2) congregational singing induces feelings of social connectedness (social effect); (3) singing religious texts is experienced as a form of prayer (singing as praying).

While the social and spiritual effects have also been mentioned in theological, liturgical, and music historical scholarship of music and singing in Catholic worship, the dispositional function has often been overlooked or subsumed under one of the others. Also, observable psychological effects have been intermixed with theological and symbolic ones (Harnoncourt, 1991). McGann (1996) states that “music carries a unique power to enable access to the experience of God and to bind persons together in community” (p. 1); while Smith (2010) maintains that music can “transform the faith lives of individual believers” by way of “deepen[ing] people’s faith” and “build[ing] community” (p. 285). Recently, Girardi (2015) has proposed that liturgical studies should start to systematically theorize and explore emotional aspects of individual experience and active participation during Mass and discussed the role of music in this regard.

In his exhaustive comparison of five papal and four other church documents on sacred music and singing in the tweenth century, Joncas (1997) also analyzes passages that mention the purposes of music in Catholic worship. He claims that the documents gradually elaborate the original double functions of music as stated in TLS as the glorification of God and “the sanctification and edification of the faithful” (Joncas, 1997, p. 32). Of these, the latter is related to the psychological and measurable effects that are of interest to the present paper. In MS, this is further differentiated into what Joncas (1997) calls the “unifying function” (which equals what is called here the social effect) and the “transcendental function” (spiritual effect) (pp. 40–41). He also comments on the passage in TLS about the dispositional effect (Joncas, 1997, p. 32); however, he does not see this as a separate effect, but rather subsumes it under edification.

### Empirical Studies of Singing Experiences and Effects

While the effects of (group) singing have so far attracted researchers from various fields, such as music psychology, social psychology, music pedagogy, or health and wellbeing, the religious contexts of communal singing are almost absent from this research (Sloboda, 2004; Belzen, 2013). Only in theology and religious studies do a few relevant qualitative studies exist. We will begin our literature review with those qualitative studies before we report on the body of general psychological studies concerning singing in order to see how far their results might apply to a worship situation.

#### Describing Religious Singing Experiences: Qualitative Studies

Qualitative studies on singing in liturgical services and religious singing events have so far been concerned primarily with various Protestant denominations (Slough, 1996; Davis, 1997; Kropf and Nafziger, 2001; Adnams, 2008, 2013; Kerner, 2008; Kinney, 2010; Kaiser, 2017). There appear to be only two studies with a Roman Catholic focus (McGann, 1996; Smith, 2010). These typically utilize methods such as participant observation, ethnography, semi-structured interviews, and content analysis of written reports. Their most valuable result is the rich and detailed description of subjective singing experiences in various religious and musical contexts, which strongly support the assumptions of Roman Catholic liturgical doctrine. In Smith’s (2010) study of music and lived faith experiences in an Australian Catholic parish, the spiritual effects of music played the most crucial role. Dispositional and social effects were mentioned to a lesser degree (although with different terminology).

Based on field research in a Canadian evangelical church, Adnams (2008, 2013) has provided a much more comprehensive typology differentiating between two dichotomous categories: “just singing” and “really worshipping.” To the “just singing” category belong sub-categories such as “un-minded singing,” “meaningless words,” and “dispassionate singing” (Adnams, 2013, pp. 187–190); these refer to modes of singing in which the singers correctly reproduce the words and the melodies, but in a mere mechanistic manner without any internal participation. They neither pay conscious attention to the words, nor succeed in making them their own spiritually or emotionally. The “really worshipping” experiences, by contrast, include experiences such as “feeling the words,” “familiar words,” and “my song is given to God” (Adnams, 2013, pp. 190–197). Here, according to Adnams (2013), “what is sung is what is felt to be real and expressed authentically in and as worship” (p. 197).

If projected onto the Roman Catholic theories about liturgical singing as laid out earlier, Adnams found primarily spiritual and dispositional effects. Social effects did not play a role in his analyses. However, his reports about “just singing” experiences should caution the churches against any belief that singing works quasi-automatically, without the involvement of situational and dispositional factors.

An even broader perspective has been taken by Kaiser (2017) in his ethnography of singing in the context of German Protestant congregations. He studied religious, aesthetic, communicative, social, and psychological dimensions of communal singing with a multifaceted methodological approach, conducting what he elsewhere termed “experience-based song analysis” (Kaiser, 2014). Although his main methods were qualitative, in several situations he also administered self-designed quantitative questionnaires with 11 items on bipolar scales that served to assess individual experiences of hymns with regard to four of the five dimensions. Thus, he was able not only to corroborate earlier findings, but also to identify five types of religious singing experiences with the help of a cluster analysis (Kaiser, 2017, pp. 409–437). Of these experience types, three were mostly positive and two neutral to negative. The positive types mainly differed along two dimensions that can be interpreted as arousal and the social dimension (Kaiser, 2017, p. 415).

The emphasis that Kaiser puts on the social dimension of congregational singing provides a valuable addition to Adnams’ earlier work. The limited extent to which the spiritual experiences are explicitly reported or deducible from his data, however, not only strengthens Adnams’ caution against the belief that music can automatically generate spiritual experiences, but may also point to cultural and confessional differences concerning how people speak about their own spiritual experiences.

#### Understanding the Effects of Group Singing: Psychological Studies

Psychological research on the effects of group singing, conducted primarily with amateur choir singers, was initiated only in the early 2000s. The relevant studies have already been summarized in several review articles (Clift et al., 2010; Clarke and Harding, 2012; Kang et al., 2017; Bullack et al., 2018). Intervention studies that compare either different measures before and after an intervention or different interventions in terms of efficacy suggest a broad range of benefits for physical, mental, and social wellbeing in not only healthy, but also ill, old, or socially excluded individuals. As physiological mechanisms that mediate such effects, the respiratory, cardiovascular, and hormonal consequences of the physical activity of singing have also been discussed (Tarr et al., 2014; Kang et al., 2017).

##### Social effects

The Church’s claim that congregational singing has a unifying effect is immediately attested by a group of studies that have shown group singing to facilitate social bonding (Kreutz, 2014; Pearce et al., 2015) and lead to feelings of social connectedness (Bullack et al., 2018) or social participation (Dingle et al., 2013), irrespective of whether group members know each other or not. However, the effects of group size (Weinstein et al., 2016) and the particular performative context, e.g., rehearsal vs. performance (Fancourt et al., 2015), are also relevant in this regard.

##### Spiritual experiences

Empirical evidence for spiritual effects is less straightforward. In general, the psychology of religion is aware of the widespread role that music and singing play in group worship practices and as a common trigger of religious experiences (Beit-Hallahmi and Argyle, 1997). There is also a rich body of qualitative research that links music (both within and outside the context of religious rituals) to religious experiences (Maslow, 1970; Greeley, 1975; Rouget, 1985; Lowis, 1998; Gabrielsson, 2011). Quantitative studies or experiments, however, have only started to emerge. While some of these studies examine the context of listening to religious music (Lowis and Hughes, 1997; Atkins and Schubert, 2014), a few others address religious experiences in the context of singing (Hills and Argyle, 1998; Clift and Hancox, 2001; Miller and Strongman, 2002). In these studies, questionnaires were used to ask members of churches and/or choirs about their singing experiences. Hills and Argyle (1998), but also Clift and Hancox (2001), found basic similarities between religious and musical experiences, with both including a social and spiritual factor. The only study so far that has quantitatively addressed religious singing experiences in the context of a specific Christian denomination is that of Miller and Strongman (2002). They examined religious experiences during the musical part of a Pentecostal-charismatic church service in New Zealand. In the first part of their study, they report the results of a questionnaire that asked about beliefs and experiences regarding music in church. The experience-related items touched exclusively on individual spiritual experiences (such as “experiences of the Holy Spirit,” “intense religious experiences,” “feeling a change in my own spirit”), and participants mostly agreed with them. Social or dispositional experiences were not considered.

##### Interaction of lyrics and music, emotional contagion

No quantitative studies could be found that address what is called here the dispositional effect in a religious context. There is, however, some research on the underlying question, i.e., how the musical and textual elements of songs interact with each other. For the liturgical context, research exploring emotional responses and research on motivational effects are both relevant. However, the existing study designs are incompatible and their results inconsistent: Stratton and Zalanowski (1994) and Sousou (1997) studied effects on mood states, i.e., felt emotion, while Ali and Peynircioğlu (2006) looked into perceived emotions. The first of these found that song lyrics had a greater effect than melody. However, the authors used only a single song as a stimulus, which, in addition, was characterized by a certain mismatch between the emotional content of the lyrics and the music. The two other studies, by contrast, found melodies to be more dominant both in conveying the intended emotion (Ali and Peynircioğlu, 2006) and in eliciting a congruent mood state (Sousou, 1997). In a sense, the much larger body of research concerning emotional responses to listening to music is relevant here as well, particularly research investigating emotional contagion effects (Juslin, 2013). Another approach was adopted by Galizio and Hendrick (1972), who explored the effects of the musical accompaniment of a text on persuasion and found that when lyrics were combined with melodies, the resulting emotional state was more positive and acceptance for the conveyed message greater. The applicability of these studies to a liturgical context, however, is limited: They only explore situations of listening, not singing, and only some cases compare text with and without music, rather than music with and without text (Stratton and Zalanowski, 1994; Sousou, 1997).

#### Aims of This Study

Although the existing quantitative studies on group singing and other musical effects that are relevant to Catholic worship point to the plausibility of the Church’s assumptions regarding congregational singing, they do not yet provide a clear and coherent picture. Concrete operationalizations of the intervention “singing” typically differ vastly from each other, as do the study designs. The musical repertoire that is sung is not controlled for or taken into account in the analyses; group sizes are small; and the empirical and experimental methods applied do not always meet the highest standards (Dingle et al., 2019). In addition, the potential influence of musical and social context factors on observed effects are typically not taken into consideration.

Therefore, the current study aims to examine real singing experiences in Roman Catholic services today in order to understand how they correspond with the framework outlined in the Church’s normative texts on liturgy and the role of congregational singing in it. A further goal of this study was to find person-related factors that have a moderating effect on such singing experiences.

### Participants

The study was performed as on online survey^1^ in which *N* = 1996 individuals from Germany, Austria, and Switzerland participated. Study participation was voluntary and anonymous. Various methods of recruitment were used, including online via mailing lists and social networks and in print via German and Austrian church newspapers. There was no monetary compensation provided for participation. After excluding all cases with more than 15 missing values and the 5% of participants who did not report being Catholic, the final dataset contained data from 1603 participants, 962 of whom were women (60%). Participants ranged in age between less than 20 and over 80 years old, the majority being between 41 and 70 (68%); and in general, they were highly educated: While 22% reported having received vocational training, 53% held a technical college or university degree and 9% a doctoral degree. Participants came mostly from the German dioceses of Cologne (30%), Mainz (22%), Trier (8%), and Rottenburg-Stuttgart (7%). Also, 65% attended Mass every Sunday and holiday, 60% in their own parish; and 91% were regular communicants. 70% performed one or more liturgical offices, such as lector (35%), extraordinary minister of Holy Communion (27%), cantor (14%), different musical offices (32%), or priest (3%); 73% engaged in other church activities as well.

With regard to congregational singing, the sample showed a very positive attitude. That they always sing in Church was reported by 80%, and 68% said that they also liked it. Even more, namely 91%, fully agreed with the statement that singing during Mass is good. In the Masses attended by 94% of the participants, the official German hymnal *Gotteslob* was used almost exclusively. Accordingly, 74% of the participants reported that they typically knew the songs that were sung in Mass. Concerning the Church’s hypothesis of the dispositional effect, it is interesting to see that 68% of the participants reported that they pay full attention to the melody of a song while singing, while only 58% said the same of the lyrics.

Participants were very much inclined toward music outside of church as well. 72% said that music and singing were personally very important for them. While 22% had no musical training, 57% had taken music lessons for at least 4 years. Also, 53% were presently musically active, 41% as members in Church choirs; and 90% also sang at least sometimes at home.

Taken together, the sample consisted mostly of highly engaged, liturgically active Catholics with a strong affinity toward music. This sample is therefore not representative of Catholics in general (of which only 9.3% attend Mass every Sunday, according to the 2019 statistics of the Deutsche Bischofskonferenz), but rather of that segment that regularly attends Mass and has thus been exposed most frequently to the stimulus in question, which is congregational singing.

### Measures

Starting from the abovementioned analysis of hypotheses underlying Church documents on the liturgy, we developed a scale based on 11 *ad hoc* formulated items that addressed social (5) and spiritual (6) experiences, the latter differentiated into anagogical experiences (4), and the idea of singing as a form of prayer (2). We did not include any items related to the dispositional effect, given the scarcity of acknowledgment or research on it so far. All items were formulated as a statement in the first person to which participants had to rate their agreement on a 5-point Likert scale from *mostly not* to *mostly yes*.

In addition, we collected data on religious and musical behavior and attitudes, as well as socio-demographics, which we will describe in more detail below. These items were chosen for inclusion as moderators or potential confounds. Again, there were no validated psychometric scales available for our purposes. Items were answered either via 5- or 7-point Likert scales that assessed degree of agreement, importance, or frequency, or by selecting one or more items from a list of response options.

#### Religious Practice

Fifteen items were used to inquire about the frequency of various types of religious behavior, i.e., frequency of attending Mass and other forms of public worship (five items), frequency of private prayer in general and in several typical forms (five items), and frequency of religious reading (five items). In addition, we asked if participants performed one or more of ten given liturgical offices.

#### Traditional vs. Secular Religious Attitude

Based on a study by Rentsch (2013), we assumed two basic understandings of the Mass, a traditional and a secular one. Ten items were created to represent either the one or the other. As far as possible, the actual wording was at least in part directly derived from the IGMR. Reflecting the traditional view, for example, were statements such as “In Holy Mass, God is acting on man” or “In Holy Mass, we become part of the heavenly liturgy into which we enter.” A secular view was represented by statements such as “In the readings and the sermon of the Mass, the faithful are asked to act for the benefit of their neighbors, just like Jesus.”

#### Religious Motivation

Participants had to rate their agreement with five items that listed possible reasons to attend Mass, as well as eight items that stated possible reasons for praying, some of which were more extrinsic (such as “Because it’s just normal” or “Because I was taught to do it”) and others intrinsic (such as “Because it is important to me and means something for me” or “Because this way, I can feel that God is near me”).

#### Musical Practice

The active musical practice of participants was assessed with four items such as “Are you currently a member in a choir?” or “Do you play an instrument?” (no/yes). In addition, four items asked about the frequency of singing at home and with regard to religious and non-religious repertoire with a 3-point Likert scale.

#### Attitude Toward Singing

To assess whether participants had more of a negative or a positive attitude toward singing, five items were created that asked about their opinion regarding singing in Mass and in private.

Sociodemographic variables included gender, age, nationality, marital status, education level, state of residence, diocese, and size of domicile, and finally, size of domicile during childhood.

### Data Processing, Reliability Checks, and Construction of Scores and Indices

Because of the exploratory nature of the study and the need to rely mostly on self-designed scales and measurements, we inspected the collected data thoroughly with regard to distribution, correlations, and underlying structure of items. We also conducted several reliability checks. Thus, we sought to reduce the number of potential predictors by either excluding or combining individual items into scores and indices. Items showing little to no variance (criterion: > 80% of responses on only one value) were excluded from further analyses.

For reliability analysis of the scale for the dependent variables, Cronbach’s alpha was calculated to assess the internal consistency of the three subscales, resulting in α = 0.79 for *Social experience*, α = 0.80 for *Spiritual experience*, and α = 0.71 for *Singing as praying*. One item, however, had to be rearranged *post hoc*: “I have the feeling that, when singing, I address God personally.” It turned out that it fitted better in the scale for *Singing as praying* than *Spiritual experience*, increasing internal consistency (from α = 0.61 to α = 0.71).

From the large number of items regarding religious and musical attitudes and behaviors we created composite scores and scales. Our criteria for combination and exclusion were face validity, reliability, and sufficient variance of the resulting scores.

#### Religious Practice

Internal consistency of the three subscales (Frequency of public worship attendance, Frequency of private prayer, Frequency of religious reading) was checked and yielded good results for the subscales *Private prayer*, α = 0.79, and *Religious reading*, α = 0.82. The reliability of the *Public worship* scale increased from α = 0.51 to α = 0.70 only after two items had been excluded. As we did not want to lose frequency of attending Mass as a single item predictor, we finally decided to combine the other items on public and private worship into a single *Worship* scale with α = 0.83. For each participant, a mean was computed in order to serve as index value. According to a comparison of the histogram of the resulting index with a normal probability curve, it was almost normally distributed (see Table 1 for descriptive statistics).

**TABLE 1:** Descriptive statistics for 12 aggregated independent variables.

Indices	*M (SD)*
**Religious practice**	
Mass attendance (Mass)	3.72 (0.99)
Frequency of worship (Wor)	2.63 (0.85)
**Religious attitude**	
Traditional understanding of Mass (Trad)	3.9 (0.85)
Secular understanding of Mass (Sec)	4.11 (0.56)
**Religious motivation**	
Intrinsic motivation (Intr)	4.23 (0.64)
Extrinsic motivation (Extr)	2.04 (0.94)
Social motivation (Soc)	3.76 (0.88)
**Spiritual experiences (SpirEx)**	3.12 (0.79)
**Musical practice**	
Choir and/or instrument^a^ (Ch/Instr)	2.04 (1.45)
Singing at home^b^ (SingH)	2.02 (0.51)
**Attitude toward singing**	
Singing in Mass (SingM)	4.72 (0.51)
Own singing (SingO)	3.68 (1.02)

*If not stated otherwise, underlying rating scales ranged from 1 to 5. ^a^Sum score from 4 items. ^b^Scale range: 1–3.*

Reports about performing one or more liturgical offices were recoded into a categorical variable which differentiated between no office, musical office, and other office.

#### Religious Attitude

The original items were designed with the intention to capture two distinct attitudes toward Mass (a traditional and a secular one; Rentsch, 2013). Internal consistency was only good for the subscale *Traditional attitude* with α = 0.87, but not for the other, α = 0.54. For theoretical reasons, however, we decided to continue with both subscales. Again, a mean for each subscale and participant was computed. The resulting indices were strongly (traditional) and slightly (secular) left-skewed, but still showed a sufficiently broad variance (see Table 1 for descriptive statistics). They were moderately positively correlated with each other (*r* = 0.44^∗∗^).

#### Religious Motivation

The related items addressed various motivations for engaging in religious practices. Since we had no *a priori* assumption of their potential grouping, we conducted a principal component analysis with Varimax rotation to see if the items would group into distinct factors. On the basis of the criterion of eigenvalues > 1, a four-factor solution was found that explained 65.7% of the variance. Two items had cross-loadings of > 0.4 on a second factor. The first and second factors combined eight items that expressed either a motivation to be near to God or the hope that Mass or private prayer might be beneficial for the individual. Three items loaded onto both factors. Therefore, we merged both factors into a subscale called *Intrinsic religious motivation* that showed a good internal consistency, α = 0.88. The third factor comprised three items of a more extrinsic motivation for private prayer that had an acceptable internal consistency of α = 0.70. The fourth factor consisted of three items that could be described as social motivation for attending Mass and was therefore kept, despite the poor internal consistency of α = 0.59. For all three factors, a mean for each participant was computed. The resulting scores were weakly to moderately associated with each other (*Intrinsic* and *Extrinsic motivation*: *r* = 0.20^∗∗^; *Intrinsic* and *Social motivation*: *r* = 0.30^∗∗^; *Extrinsic* and *Social motivation*: *r* = 0.17^∗∗^).

#### Religious Experience Outside Mass

We created a scale for frequency of spiritual experiences independent of singing in Mass from four items that focused on the frequency of experiences of being in close contact or communication with God. The internal consistency of this scale was good, with Cronbach’s α = 0.81. For each participant, a mean score from these four items was created.

#### Musical Practice

The active musical practice of participants was assessed by counting to how many of the four related items they had answered with “yes.” In addition, we computed a mean for each participant from the four items on frequency of private singing at home.

#### Attitude Toward Singing

The internal consistency of a scale consisting of the four items on attitudes regarding singing in Mass and on one’s own was not satisfying, Cronbach’s α = 0.67. A principal component analysis with Varimax rotation revealed two underlying factors that explained 65.7% of the variance, each consisting of two items. From these, a mean score per factor and participant was computed. The first score included two items on singing in Mass, the other on participants’ own singing. The association of the two scores had a moderate strength (*r* = 0.32^∗∗^).

The relationships between the practice and attitude variables can be seen in the correlation matrix in Table 2. The most notable patterns are the moderate to strong associations between three of the religious practice variables (i.e., frequency of attending Mass, worshipping, and spiritual experiences) and two religious attitude variables (i.e., traditional understanding of Mass and intrinsic motivation to attend it) on the one hand, and musical practices and attitudes toward singing on the other.

**TABLE 2:** Correlations between religious and musical practices and attitudes.

	Religious Practice				Musical practice			
Attitude	In Mass		Outside Mass		In Mass		Outside Mass	
	Mass	LitOff	SpirEx	Wor	MusOff	FrSing	Ch/Instr	SingH
Trad	0.51**	0.15**	0.48**	0.55**	0.01	0.07**	−0.05*	0.10**
Sec	0.14**	0.11**	0.27**	0.09**	0.04	0.13**	0.00	–0.00
Intr	0.32**	0.14**	0.58**	0.39**	−0.06*	0.10**	−0.09**	0.09**
Extr	0.08**	0.07**	0.08**	0.06*	0.02	0.04	–0.00	–0.02
Soc	0.11**	0.09**	0.13**	−0.05*	0.14**	0.29**	0.17**	0.14**
SingM	–0.01	–0.02	0.04	−0.09**	0.17**	0.59**	0.25**	0.18**
SingO	0.05*	−0.18**	0.07*	0.04	0.35**	0.37**	0.52**	0.46**

Abbreviations of indices as introduced in Table 1. In addition: LitOff, performance of non-musical liturgical office; MusOff, performance of musical liturgical office; FrSing, frequency of singing along in Mass. *p < 0.05. **p < 0.01.

## Results

### Prevalence of Singing Experiences

Inasmuch as the main aim of this study was to test whether congregational singing in Catholic worship contexts today could generally afford the social and spiritual experiences that the Church expects, we first examined the means and distributions of the related items (see Table 3).

**TABLE 3:** Item battery and descriptive statistics for effects of congregational singing in mass.

Item	*M (SD)*	Kurtosis	Skewness
**Social experience**			
I sing together with the others.	4.4 (0.8)	1.9	–1.4
I feel connected with the others while singing.	4.1 (0.9)	0.4	–0.9
Even if I do not sing along, I feel connected with the others when they sing.	3.6 (1.2)	–0.6	–0.6
I have the feeling that we become a community when we sing in Mass.	4.2 (0.9)	1.1	–1.1
I feel supported by the others’ singing.	3.6 (1.1)	–0.5	–0.4
**Spiritual experience**			
When singing, I have the feeling of being close to God.	3.7 (1.0)	–0.5	–0.4
For me, singing in general expresses a bond between us congregants and Heaven.	3.7 (1.1)	–0.5	–0.6
For me, particular songs express a bond between us congregants and Heaven.	3.9 (1.1)	–0.1	–0.8
**Singing as praying**			
Singing in community supports my own prayer in Mass.	4.1 (1.0)	0.9	–1.1
I have the feeling that, when singing, I address God personally.	3.8 (1.1)	–0.3	–0.6
I experience my own singing as supportive of the others’ prayer in Mass.	3.5 (1.2)	–0.9	–0.4

*N = 1603; means based on a 5-point Likert scale.*

The means of the 11 items with liturgy-relevant effects all lay above the neutral middle point of 3, ranging from 3.5 (*SD* = 1.2) for “I experience my own singing as supportive of the others’ prayer in Mass” to 4.4 (*SD* = 0.8) for “I feel connected with the others while singing.” All distributions were (strongly) left-skewed and differed significantly from the neutral scale middle [*t*(1565–1596) = 17.3–71.6, all *p* < 0.001, *d* = 0.9–3.6]. In eight cases, the highest scale point was also the most frequently chosen value. Of all three, the social effect was experienced most frequently.

### Predictors for Singing Experiences

In a next step, we examined statistical relationships between our independent measures and the effects of congregational singing in Mass. First, three separate linear multiple regression models were fitted for each effect using SPSS Version 25 (method: inclusion), which included one of the three predictor types—practices, attitudes, and sociodemographic variables—in order to gain an overview of their relative strengths.

In the models with sociodemographic variables, the predictors we included were age, gender, level of education, and size of domicile during childhood and at present. The models with practice variables included Frequency of attending Mass, Performing liturgical office (musical and other), and Frequency of singing in Mass, as well as the indices for Frequency of worship, Musical practice in choirs or ensembles, and Singing at home. The models with attitude variables consisted of the indices for Religious attitude, Religious motivation, Spiritual experiences, and Musical attitudes.

Both practices and attitudes significantly predicted singing experiences in Mass to a sufficient degree; sociodemographic variables, however, did not (see Table 4).

**TABLE 4:** Model summaries.

	Effect		
Models	Spiritual experience	Singing as praying	Social experience
Sociodemographic	*F*(5,1597) = 1.24	*F*(5,1597) = 3.84**	*F*(5,1597) = 8.36***
variables		adj. *R*^2^ = 0.01	adj. *R*^2^ = 0.02
Practice variables	*F*(7,1595) = 34.60***	*F*(7,1595) = 53.91***	*F*(7,1595) = 20.56***
	adj. *R*^2^ = 0.13	adj. *R*^2^ = 0.19	adj. *R*^2^ = 0.08
Attitude variables	*F*(8,1594) = 114.83***	*F*(8,1594) = 120.33***	*F*(8,1594) = 79.31***
	adj. *R*^2^ = 0.36	adj. *R*^2^ = 0.37	adj. *R*^2^ = 0.28
Practice and attitude	*F*(3,1599) = 267.65***	*F*(4,1598) = 203.15***	*F*(2,1599) = 227.75***
variables	adj. *R*^2^ = 0.33	adj. *R*^2^ = 0.34	adj. *R*^2^ = 0.22

**p < 0.01. ***p < 0.001.

Nonetheless, not all of the variables included turned out to be significant predictors. In the models with practice variables, Musical liturgical office and Musical practice in choirs or ensembles did not significantly predict Spiritual experience, while Frequency of Mass attendance, Frequency of participation in public and private worship, Musical practice in choirs or ensembles, and Singing at home had no predictive value for Social experience. In the models with attitude variables, single items or indices that were not able to predict effects were Secular understanding of Mass (for Spiritual experience and Singing as praying), Intrinsic religious motivation (for Social experience), Extrinsic religious motivation (all three models), and Spiritual experiences (for Social experience).

To see how practice and attitude variables would behave if combined in one model, we fitted three further models with both types of predictors. However, to avoid multicollinearity, predictors with correlation coefficients of *r* > 0.40 to other predictors that themselves were stronger correlated with the dependent variables were excluded beforehand. This time, we decided for stepwise inclusion as a method in order to identify the most relevant predictors. The results are presented in Tables 4, 5.

**TABLE 5:** Standardized beta coefficients of practice and attitude predictors for three effects of congregational singing in mass.

	Effect		
Predictor	Spiritual experience	Singing as praying	Social experience
**Religious predictors**			
Traditional understanding of Mass	0.45***	0.37***	
Secular understanding of Mass			0.22***
Social religious motivation			0.35***
**Musico-religious predictors**			
Attitude toward singing in Mass	0.27***	0.30***	
Musical office		0.10***	
**Musical predictors**			
Singing at home	0.14***		
Attitude toward own singing		0.16***	

***p < 0.001. To avoid multicollinearity, predictors with correlation coefficients of r > 0.40 to other predictors that themselves were stronger correlated with the dependent variables were excluded.

When practice and attitude variables were combined, but multicollinearity rigorously reduced, only two to four predictors survived, thus reducing the overall goodness of fit of the models somewhat compared to the models with attitude variables only. In the models for Spiritual experience and Singing as praying, one musical practice predictor (Singing at home and Musical office respectively) complemented two to three attitude predictors, the two strongest of which appeared in both models (i.e., Traditional understanding of Mass and Attitude toward singing in Mass). The final model for Social experience included only two religious attitude predictors which were different from those in the other models.

Practice variables that appeared as significant predictors in the practices-only models but disappeared from the combined models were: Frequency of participation in public and private worship, Frequency of singing in Mass, and Performance of a non-musical liturgical office. Frequency of participation in public and private worship was strongly associated with Traditional understanding of Mass (*r* = 0.53^∗∗^). Frequency of singing in Mass was strongly associated with Attitude toward singing in Mass (*r* = 0.61^∗∗^) and also moderately associated with Attitude toward one’s own singing (*r* = 0.38^∗∗^) and Social religious motivation (*r* = 0.28^∗∗^). Performance of a non-musical liturgical office was also associated with a number of attitude variables, but only weakly (no *r* > | 0.18|). Except for the case of Performance of a non-musical liturgical office, it seemed quite clear that the two other practice variables were the behavioral consequences of related attitudes, which is why they no longer became significant on those models that included the attitudes underlying them.

In sum, religious and musico-religious attitudes were by far the strongest predictors of Mass-relevant experiences of congregational singing. While the models for Spiritual experience and Singing as praying shared their two strongest predictors and were also similar, inasmuch both contained a musical practice predictor, Social experience showed a distinct pattern being related to a secular understanding of Mass and a social motivation to attend it.

## Discussion

The Roman Catholic Church has always expected a lot from sacred music in its liturgy—as have other Christian denominations and many other religions. In the wake of the reforms initiated by the Second Vatican Council, chants and hymns sung by the congregation have been assigned the function of causing attendants to realize, feel, and experience individually what is performed and enacted in the liturgy. But can congregational singing in Catholic Masses actually and reliably afford a majority of congregants with the social and spiritual experiences envisaged in the Church’s authoritative documents? In this study, we have for the first time provided quantitative data on this question from a large German-speaking sample. We have found that congregational singing is indeed very frequently experienced as having the unifying and uplifting effects on which the Church has based its liturgical practices regarding singing. We also found that the degree to which these effects are experienced largely depends on religious and musical attitudes.

### Summary and Interpretation of the Findings

#### Affirmation of Hypotheses

Our study was conducted as an online survey, which featured an exhaustive questionnaire about three types of general singing experiences in Catholic Masses and a broad range of potentially relevant factors, including religious and musical practices and attitudes. The singing experiences that were asked for were derived from the Church’s current, post-conciliar theory and theology of Mass (SC, MS, IGMR), namely feeling of community (social effect), feeling of being close to God (spiritual effect a), and experiencing singing to be a form of prayer (spiritual effect b).

We have found that congregational singing in Mass can indeed afford these effects to a large degree, whereby the social effect is even more pronounced than the other two—at least for a sample like ours, which was composed mostly of well-educated regular churchgoers with a strong affinity for music. Thus, the theoretical assumptions, historical documents, and qualitative data from earlier studies of worshippers from various Christian denominations (Slough, 1996; Adnams, 2008; Kaiser, 2017) have been corroborated by our quantitative data. Our results are also consistent with the existing body of qualitative and quantitative research on group singing as an instrument for social bonding (Kreutz, 2014; Pearce et al., 2015; Bullack et al., 2018).

Although there exists very little quantitative research on the spiritual effects of music and (group) singing, the findings so far point in the same direction as ours and show that people do have spiritual experiences in conjunction with music (Hills and Argyle, 1998; Clift and Hancox, 2001; Atkins and Schubert, 2014; Demmrich, 2018). Specifically, the studies by Hills and Argyle (1998) and Clift and Hancox (2001) have shown that group singing is associated with several experienced effects or benefits simultaneously, including social and spiritual ones.

#### Explanation of Effects

We also looked into potential intraindividual predictors. Given that we could not formulate hypotheses based on earlier research, we tested sociodemographic variables, religious and musical practices, and religious and musical attitudes against each other. While practices and attitudes turned out to significantly predict the frequency of liturgical singing experiences, sociodemographic factors had almost no effect at all (see Table 4). Attitudes, however, had a much stronger predictive value than practices and overwrote most of the latter in those models that included both variable types. The most important attitude variables were a traditional understanding of Mass (operationalized after Rentsch, 2013) and a positive attitude toward singing in Mass which both predicted Spiritual effects, and a social motivation to attend Mass which predicted the Social effect (see Table 5). They must therefore count as the most critical factors behind individual experiences of congregational singing in Catholic worship.

In general, the models for Spiritual experience and Singing as praying showed many similarities, but were distinct from that for Social experience. While the latter was the only one for which a secular understanding of Mass played a significant role, the two other models comprised also musico-religious and musical predictors.

Taken together, the types and combinations of predictors found to be significant in our regression models have a high face validity and are also consistent with earlier research. In the case of religious attitudes, the role of religiosity for actual spiritual experiences with music already manifested in the studies by Lowis and Hughes (1997) for listening to music and by Clift and Hancox (2001) for group singing.

The relationship between attitudes, responses, and related behavior has been studied extensively. Attitudes are known to influence individual emotional, cognitive, and behavioral responses to objects and situations (Ajzen, 2001; Ajzen and Fishbein, 2005), which is what we have found for the case of congregational singing. They also predict behavior (Fishbein and Ajzen, 1975), and do so even more the more frequently a related behavior is performed (Davidson and Jaccard, 1979; Fazio, 2000). This may account for the fact that in our case, the two behavior (=practice) variables Frequency of singing in Mass and Frequency of public and private worship were overwritten by related attitude variables in the final models. Since attitudes are known to be learned, i.e., to be at least in part the result of earlier behaviors and experiences (Fishbein and Ajzen, 1975), religious and musical practice can still be assumed to play an important, albeit indirect, role for the frequency and quality of singing experiences in Mass. Concretely, religious and musical practices, the experiences they generate, and the collective knowledge they convey can be expected to form attitudes that then in turn influence actual experiences.

This leads to the conclusion that, clearly, music in general and communal singing in particular do not function as automatic mechanisms generating social, religious, or any other experiences in people solely due to their musical and performative properties. This finding calls to mind research on music and trance. Here, as well, the original notion that there are certain kinds or elements of music that directly induce a trance state has been overcome by thorough anthropological and psychologically informed ethnomusicological research (Rouget, 1985; Becker, 2004).

Another conclusion from our findings is that singing experiences in a religious context such as a Catholic Mass not only depend on the religious context, but are informed by the sphere of music as well, since musical attitudes and practices outside Church are to a certain extent able to predict experiences during worship. The experiences afforded by the texts, music, contexts, and performance of Church songs need to meet with congruent dispositions, attitudes, preferences, and practices (in terms of religiosity and musicality) in order to come to life. This becomes particularly clear in the comparatively large *R*^2^ values of our models—given that these included effects on the individual level, but not direct effects of stimuli.

### Limitations and Future Studies

Inasmuch as it was the main aim of this study to establish, quantitatively, whether and to what extent congregational singing in Roman Catholic worship can facilitate certain experiences that are anticipated in the Church’s norms, we decided to run an online survey in order to reach out to a large number of participants from all over the German-speaking area. This approach allowed us to gauge general beliefs and cumulative experiences with singing in Mass; but at the same time, it came with the disadvantage of not involving direct responses to real stimuli in specific worship situations. Thus, we were unable to gain any information about which particular singing experiences, hymn repertoires, and settings people were internally referring to. Further, this approach led to a somewhat biased sample, particularly with regard to the keen interest in singing and music and the very positive attitudes that most participants held toward singing. Churchgoers who were less inclined to join in congregational singing or Catholics who attended Church only occasionally were much less motivated to participate in the survey. This sample bias might have led to a much too positive picture concerning the occurrence of spiritually meaningful singing experiences. Further, we only used direct measures to ask about singing experiences. Assuming that active Catholics would be more or less aware of what functions and effects congregational singing was expected to have, and also given the positive attitude toward singing in our sample, social desirability effects cannot be excluded. The high levels of skewness that our main variables showed could also point to a ceiling effect. In this case, a scale with more than five division points would be needed.

Therefore, the obvious next step would be to administer a similar questionnaire in the context of actual Masses to a more diverse group of participants in order to gather more direct responses that are less affected by memory bias. Also, musical stimuli, contextual factors (such as the number of attendees and number of worshippers who do not sing), and liturgical performance could be documented and fit into models.

This questionnaire could benefit from including not only direct measures, but also indirect and potentially even (more) objective measures of singing experiences, in order to avoid or at least to reduce social desirability effects. On the side of predictors, validated scales should be constructed and preferred over self-designed items. In addition, it would be useful to obtain liking and familiarity ratings for each hymn and chant, as they can be expected to serve as powerful moderators of main effects (see Porter, 2017, on musical tastes and individual preferences of congregants). Since the dispositional effects of singing, which can be extrapolated from the authoritative documents, were not part of the present study, but are regarded as crucial by the Church, efforts should be made to find a well-functioning operationalization and to include this in the questionnaire. Another conceivable option for addressing the dispositional effect would be to design an experiment that directly tests whether and under what circumstances a musical setting of a text can enhance the emotional and motivational effect of the text itself.

A further limitation of the present study is that it is bound to the liturgical style of a particular Christian denomination; therefore, generalizability is only possible to a minimal degree. At the same time, this might also be considered an advantage: Although the various denominations expect congregational singing to have comparable effects, and although many primarily qualitative studies have shown social, spiritual, and dispositional effects in non-Catholic worship contexts as well, the actual forms, repertoires, extent, and liturgical role of congregational singing differ tremendously between denominations and cultural areas.

### Implications and Conclusions

The present study has immediate and obvious implications for the Catholic Church, for theorists and norm-givers of liturgy, but even more so for liturgical practitioners such as priests, cantors, and church musicians. They can be reassured that, in principle, congregational singing in Catholic worship can have the desired effects, aiding the spiritual efficacy of the divine service. However, they should also acknowledge the fact that individual religiosity and musicality play a considerable role as well. In addition, our study can be taken as an encouragement for liturgical and hymnological studies to integrate quantitative and experimental methods into their scholarly toolkit.

Outside the scope of the Church, our research adds to the growing body of psychological research on the effects of group singing, focusing on a specific socio-cultural context. We further support the already well-established assumption of collective music-making as a facilitator of social bonding in the specific context of Catholic congregations. We have contributed to the so far scarce quantitative evidence on singing experienced as a religious activity. At the same time, our study goes far beyond existing qualitative and quantitative studies with regard to the large number and types of independent variables included. Thus, we were able to find meaningful influencing factors on the level of religious and musical attitudes, factors that had not been examined before.

From a theoretical perspective, our found predictors must be interpreted as moderators of the primary effects of stimuli, i.e., the sung hymns and chants, together with their respective liturgical performances and settings. The relatively strong predictive value of these moderating factors, however, points to the fact that the psychological effects of music, more specifically of communal singing, that we have studied in this paper, are not so much intrinsic to the songs and singing themselves, but are to a large degree dependent on prior experience, predisposition, preference, and participation in a shared interpretative frame. In other words, a culturally formed behavior such as singing religious songs discloses higher-level effects and meanings only within its respective cultural context. This message needs to be taken (more) seriously not only by the Catholic Church, but also by certain approaches in music psychology that tend to naturalize and essentialize the large variety of humanly organized sound and related practices into the generic term of music.

## Data Availability Statement

The raw data supporting the conclusions of this article will be made available by the authors, without undue reservation.

## Ethics Statement

The studies involving human participants were reviewed and approved by the Ethics Committee of the Max Planck Society. The patients/participants provided their written informed consent to participate in this study.

## Author Contributions

MW-F, SB, and KD contributed to the conception and design of the study, identified the hypotheses, and designed the questionnaire. TV and MW-F performed the statistical analysis. MW-F wrote the manuscript. All the authors contributed to manuscript revision, read, and approved the submitted version.

## Conflict of Interest

The authors declare that the research was conducted in the absence of any commercial or financial relationships that could be construed as a potential conflict of interest.

## Footnotes

1 www.unipark.com
